# Evaluation of a Collaborative Telehealth Model for Eye Care Between Ophthalmology and Optometry in Western Australia

**DOI:** 10.1111/ajr.70203

**Published:** 2026-05-07

**Authors:** Jingyi Chen, Khyber Alam, Stephen E. Bartnik, Sharon A. Bentley, Allison M. McKendrick, Sandra C. Thompson, Angus W. Turner

**Affiliations:** ^1^ Department of Optometry and Vision Sciences, School of Health and Clinical Sciences The University of Western Australia Crawley Western Australia Australia; ^2^ Oklahoma College of Optometry Northeastern State University Tahlequah Oklahoma USA; ^3^ Lions Outback Vision Broome Western Australia Australia; ^4^ Herbert Wertheim School of Optometry and Vision Science University of California Berkeley Berkeley California USA; ^5^ Lions Eye Institute Nedlands Western Australia Australia; ^6^ Western Australian Centre for Rural Health The University of Western Australia Geraldton Western Australia Australia; ^7^ Centre of Ophthalmology and Visual Science The University of Western Australia Nedlands Western Australia Australia

**Keywords:** collaborative, digital health, ophthalmology, optometry, telehealth, teleophthalmology, workforce

## Abstract

**Objective:**

Recent advances in technology and the impact of COVID‐19 have expanded the adoption of digital health. Synchronous collaborative telehealth between optometry and ophthalmology has been used to expedite specialist eye care in Western Australia since 2011. Optometrists perform an in‐person assessment and participate in videoconferencing with ophthalmology to facilitate shared decision‐making. This study utilises existing implementation theory to assess factors influencing implementation success, sustainability and scalability of this telehealth model.

**Setting:**

Regional, rural, and remote Western Australia.

**Design:**

In‐depth interviews were undertaken using an interview guide based on the Consolidated Framework for Implementation Research (CFIR). Deductive analysis was used to code themes to the framework, and inductive analysis was used for themes relating to sustainability and scalability.

**Participants:**

Sixteen clinical and non‐clinical staff with experience in telehealth for eye care in rural Western Australia.

**Results:**

Key themes identified using the CFIR included the relative advantage of telehealth (innovation), optimising workflow and role clarification (implementation process), funding (external setting), technological infrastructure (inner setting), a champion, and ownership and adaptability (individuals). Stakeholder buy‐in, resource allocation, integration into public health infrastructure, asynchronous telehealth, and artificial intelligence were themes pertaining to both sustainability and scalability.

**Conclusion:**

This study proposes key considerations in the implementation and maintenance of a collaborative telehealth model for eye care in rural areas which may be used as a basis for guideline development or to replicate the model in other contexts.

## Summary

**What is already known on this subject?:**

- Collaborative telehealth between ophthalmology and optometry can improve access to specialist services in rural areas.
- Telehealth allows direct surgical bookings to bypass the hidden waiting list, reduces wait times, reduces patient transfers to the city and improves efficiency of visiting ophthalmology services.
- Collaborative telehealth has been used in rural Western Australia but the factors influencing the implementation, sustainability and scalability of this model were not well understood.

**What does this study add?:**

- Key factors that have influenced implementation success of this model include optimising workflow and role clarification for practitioners, funding, technological infrastructure, having a champion, and adaptability of team members.
- Effective stakeholder engagement, integration into the public health system and utilisation of asynchronous telehealth and artificial intelligence are important factors for sustainability and scalability and may further increase the impact of collaborative telehealth for eye care.

## 1 Introduction

Internationally, eye care needs are predicted to rise with the ageing population [1]. In Australia, there is a shortage of ophthalmologists, with the majority working in private metropolitan settings [2]. There is high demand placed on public ophthalmology, and wait times for public cataract surgery in Australia remain one of the longest among developed countries [3, 4]. Access to care issues are compounded for rural residents due to limited services and workforce maldistribution. Delayed care for chronic conditions such as glaucoma can lead to significant personal and economic costs [5, 6]. Therefore, it is necessary to explore alternative ways to deliver eye care.

Telehealth has been used in several health professions to expedite and increase access to care. Whilst initial uptake was variable and slow [7], the COVID‐19 pandemic and advances in technology have fast tracked the recent use and uptake of telehealth for eye care [8]. There is a need to evaluate modern models of telehealth in eye care to understand their effectiveness and potential scalability.

In rural and remote Western Australia, 540 000 people live across more than 2 million square kilometres of land. Visiting ophthalmologists historically serviced this widely dispersed population; however, services were limited in frequency and wait times were long [9]. To address these challenges, Lions Outback Vision (LOV) has used collaborative telehealth between optometry and ophthalmology since 2011 to increase access to public ophthalmology services. This model has received positive patient feedback and acceptance [9].

While both the optometry and ophthalmology workforce are maldistributed, the optometry workforce is larger and more geographically dispersed. The Australian optometric workforce is highly trained in eye care and well equipped with diagnostic equipment, providing a compelling economic case for ophthalmology to partner with [10]. In Australia, differences in funding between optometry and ophthalmology services create unique economic considerations for this interprofessional collaboration. LOV offers a public ophthalmology service which is funded by the State government and by philanthropy. Optometry in Australia is not typically part of the public health care system, and services are subsidised by Australia's national universal health insurance scheme, Medicare.

The LOV model primarily utilises synchronous telehealth, involving a single optometrist at the patient‐end engaging in video consultation with an ophthalmologist, which fulfils the reimbursement requirements from Medicare [11]. Optometrists may be in community practice, working in a hospital, or conducting outreach to remote communities. The role of the optometrist is to perform an in‐person comprehensive ocular examination and to facilitate shared decision‐making. For ophthalmology, registrars are the first point of call and are rostered to telehealth alongside a senior ophthalmologist who provides oversight. Other delivery method possibilities for collaborative telehealth for eye care, such as asynchronous, are not currently subsidised by Medicare and are not explored in this study.

LOV telehealth aims to maximise utilisation of the workforce and reduce care duplication, and is used for acute, chronic and conditions treatable by surgery [12, 13]. The goal for acute conditions is to coordinate emergency patient transfers to the city or prevent avoidable transfers by stabilising patients in the community. Inclusion of chronic or stable conditions intends to alleviate the burden on public ophthalmology and expedite care. Direct surgical bookings bypass a separate outpatient consultation, which streamlines the surgical pathway and reduces wait times [14].

The literature reports few other Australian programmes using telehealth for eye care [15, 16, 17]. Indeed, many telehealth programmes have started but ceased operating in Australia [18]. Therefore, it is important to understand the factors that have influenced initial and sustained implementation of the telehealth model in Western Australia. A primary driver for telehealth implementation is acceptance by the service deliverer [19], making this a critical perspective that is explored in this study. Other factors that influence the success and sustainability of telehealth services include having a clear vision of purpose, ownership, adaptability, economics, efficiency and equipment [20]. Many existing qualitative studies have explored clinician perspectives on barriers and facilitators to asynchronous teleophthalmology [21, 22, 23], rather than the interactive and collaborative nature of this model of telehealth.

Therefore, the objective of this study was to explore and understand the contextual factors that have influenced the implementation of a synchronous collaborative telehealth model between optometry and ophthalmology through interviews with service providers, guided by the Consolidated Framework for Implementation Research (CFIR) [24]. This study sought to identify factors that have influenced the sustained success of the model, its future sustainability and provide recommendations for replicating similar models in other contexts.

## 2 Methods

### 2.1 Ethics

This study adhered to the tenets of the Declaration of Helsinki and ethics approval was granted by the University of Western Australia Human Research Ethics Committee (2024/ET000213). All participants provided informed consent prior to participation.

### 2.2 Study Design and Participants

This study was guided by the CFIR, which is a multi‐level, well‐operationalised framework with five domains that influence implementation success (Table 1) [24]. This framework is one of the most widely utilised determinant frameworks, at pre‐ or post‐implementation phases, to explain the contextual factors surrounding implementation [24]. For this study, the CFIR informed the development of a semi‐structured interview guide (File S1) with questions pertaining to each of the five domains: innovation, outer setting, inner setting, individuals and implementation process. Beyond the CFIR, additional questions on the sustainability and scalability of the LOV telehealth model were added to the interview guide. The guide was reviewed by optometry and ophthalmology colleagues, pilot tested with three individuals, and refined based on feedback. Key informant sampling was used to invite service deliverers with current or previous professional experience and knowledge regarding this telehealth set‐up [25].

**TABLE 1:** Consolidated Framework for Implementation Research (CFIR) domains.

Domain	Definition
Innovation	The innovation itself, separate from the implementation strategy.
Outer setting	The broader environment or context in which the inner setting operates and interacts.
Inner setting	The internal environment where the innovation is implemented.
Individuals	The people involved in the innovation, including their roles and characteristics.
Implementation process	The actions and strategies employed to implement the innovation.

### 2.3 Data Collection

Semi‐structured interviews were conducted between February 2025 and May 2025, either in‐person or online using Microsoft Teams. Interviews allowed for a deep understanding of the perspectives of participants without any pressure or external influence that may arise in focus group settings. Participants were interviewed by the first author, an optometrist trained in interviewing and qualitative techniques. The interviewer had previously observed the telehealth model but had not provided any of the services herself. Prior to the commencement of the interview, participants were informed of the aims of the study to provide context. Interviews were recorded with consent, transcribed verbatim using Microsoft Teams, checked by the interviewer and deidentified. The mean duration of the interviews was 53 min (range 25–86 min).

### 2.4 Data Analysis

Data collection, transcription and analysis occurred contemporaneously. Deductive analysis was employed to chart the data pertaining to the CFIR domains. For responses pertaining to sustainability and scalability, an inductive approach was taken to allow themes to emerge. Coding was undertaken using NVivo 14 by two researchers independently; the first author and a co‐author who is also an optometrist and experienced with qualitative techniques and health systems research. During the process of analysis, the two researchers met frequently to discuss their interpretation of the data and the potential influence of their own positionality. Any differences between researchers were discussed with the broader research team to reach a consensus. Interviews were conducted until data saturation, which was the point where no new themes emerged.

### 2.5 Rigour

Several techniques were used to increase trustworthiness and rigour of this study. Data analysis by two different researchers with different backgrounds increased confirmability of results. Whilst both researchers understood the terminology and industry‐specific nuances to eye care, divergence in cultural and geographic experiences of the researchers provided greater dimensionality to the study [26]. Memoing was done to record the researchers' feelings and thoughts during data collection and analysis for dependability [27]. An audit trail enhanced credibility and dependability of the results. The context, population, and settings of participants are reported in detail to enhance transferability of the results [28].

## 3 Results

**FIGURE 1:**
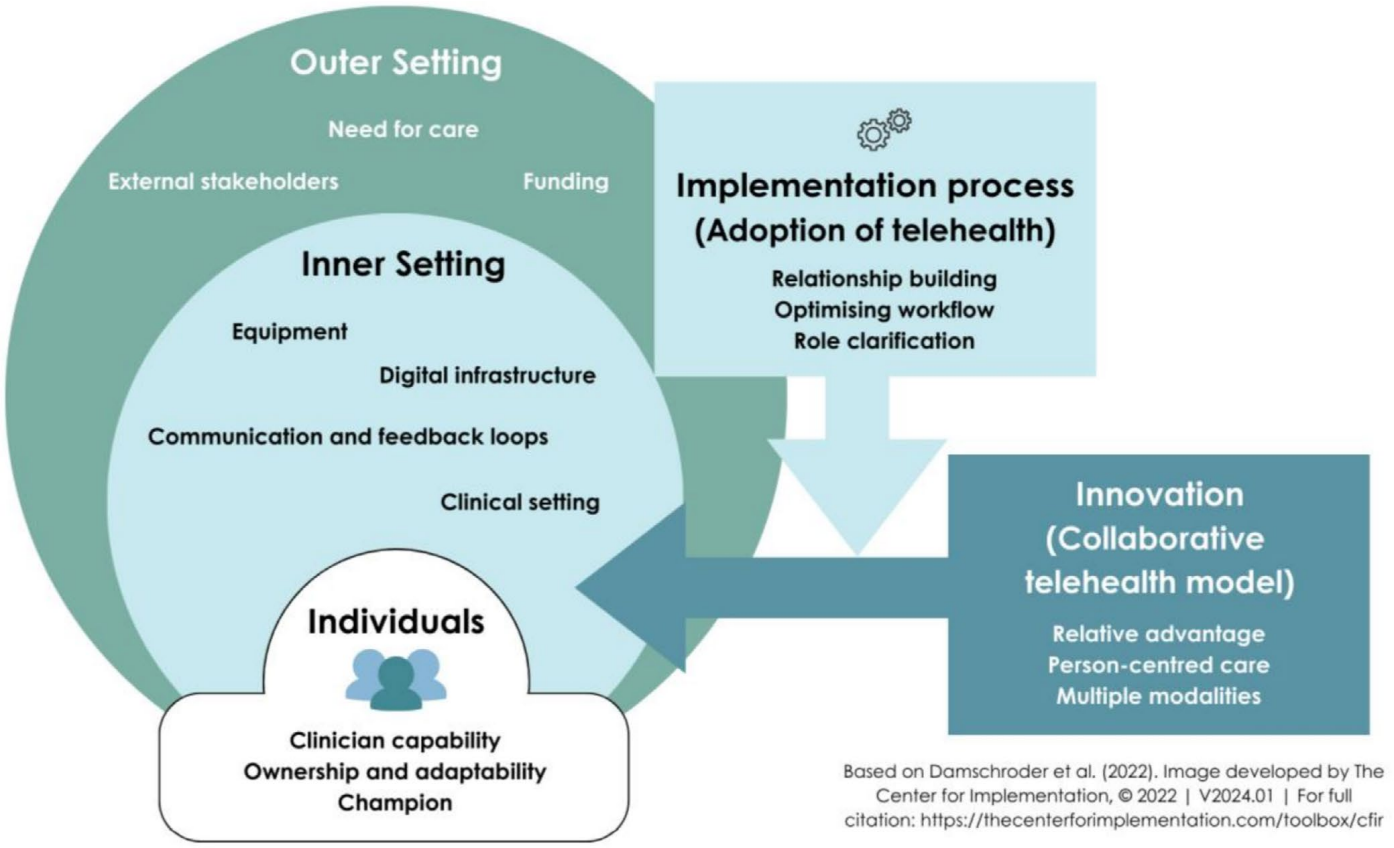
Overview of themes on the implementation of a collaborative telehealth model between ophthalmology and optometry organised according to the Consolidated Framework for Implementation Research (CFIR) [24, 29].

**FIGURE 2:**
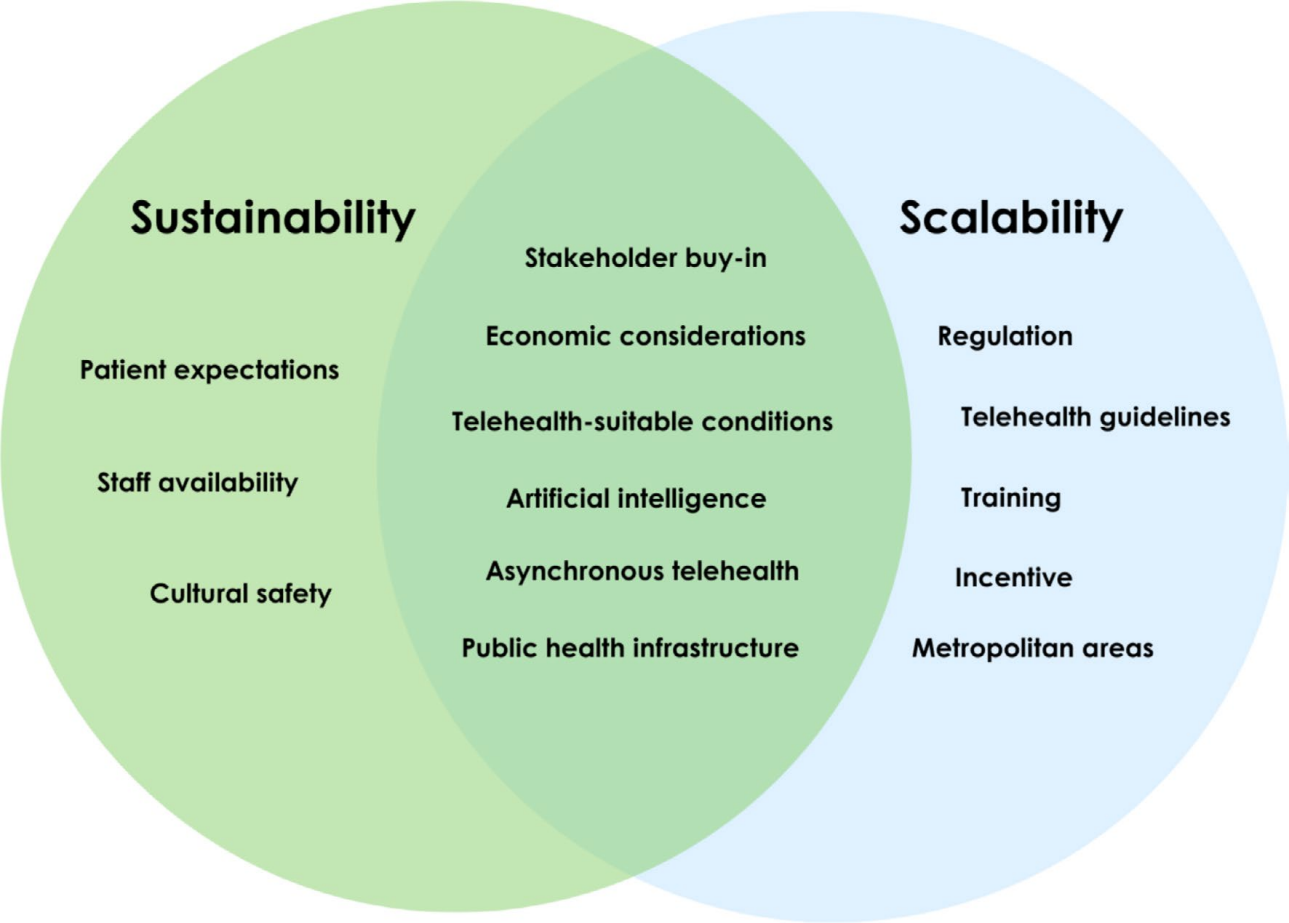
Factors influencing sustainability and scalability in a collaborative telehealth model between ophthalmology and optometry.

Interviews were conducted with 16 participants. Participants included optometrists (*n* = 5), ophthalmologists (*n* = 4), ophthalmology registrars (*n* = 5) and non‐clinicians involved in the service (*n* = 2). Non‐clinical staff had experience in management and patient liaison. All participants had recent or current experience with the telehealth model at the time of the interview. Of the 16 participants, 75% (*n* = 12) identified as male and 25% (*n* = 4) identified as female. Sixteen sub‐themes were generated relating to the five CFIR domains, as shown in Figure 1. Additionally, three sub‐themes emerged regarding sustainability, four regarding scalability, and five pertaining to both, shown in Figure 2. A summary of recommendations was developed based on the findings (Table 2).

**TABLE 2:** Recommendations for sustainability and scalability of collaborative telehealth between optometry and ophthalmology for eye care in Australia.

Area	Recommendations
Stakeholders	Engagement with professional bodies and prospective users for broader implementation.
Digital infrastructure	Usage of videoconferencing platforms that are accessible with different devices, and streamlined online booking processes. Usage of electronic medical records and interoperable platforms that enable clinical and imaging data sharing.
Guidelines	Collaborative development of evidence‐based guidelines for telehealth, including but not limited to telehealth‐suitable conditions, necessary investigations specific to conditions, and ethical, privacy and regulatory considerations.
Funding	Review of remuneration model for uplift of telehealth, including Medicare rebates, to appropriately reflect clinical care provided.
Public health infrastructure	Implementation of synchronous and asynchronous telehealth in public hospitals to expedite care and surgical bookings.
Training	Provision of support, training and collaboration opportunities for clinicians and non‐clinicians.
Incentive	Consider an ongoing incentive for optometrists and ophthalmologists for increased usage of telehealth, given cost‐effectiveness for the health system.
Asynchronous	Adoption of asynchronous modalities for stable and/or chronic conditions, in conjunction with synchronous, to increase efficiency of telehealth. Determine appropriate remuneration for asynchronous telehealth.
Data‐informed implementation	Utilise data‐informed strategies to trial telehealth in rural and urban areas with population‐based need.

### 3.1 Telehealth Model

#### 3.1.1 Relative Advantage

Much of the impetus to use telehealth arose from the perceived relative advantage of the model in the context of extremely limited access to traditional face‐to‐face ophthalmology care. Broadly, participants agreed that the purpose of telehealth is not to replace in‐person ophthalmology care, but to serve as a crucial tool for improving access to secondary eye care. Participants reported the main advantages of telehealth as reduced wait times and reduced travel. For rural residents whom this model benefits, travel could often involve hundreds or thousands of kilometres, with added complexities of caretaking duties and productivity loss from time off work. Other advantages included continuity of care due to the optometrist being local and reducing the burden on public ophthalmology through appropriate resource allocation, that is, face‐to‐face ophthalmology services can be reserved for those who require it.

#### 3.1.2 Person‐Centred Care

Above all, person‐centred care was the priority in care provision. One optometrist felt that patients ‘feel engaged, they get to meet and view the surgeons, there's a transference of trust between primary and secondary care. It's a win‐win situation’ (OPTOM2). Patient safety was paramount; ‘if we're finding that we're not able to get enough information to make an appropriate decision, then we just go with getting the patient to Perth or booking them in for our next [in‐person] clinic…So you maximise the benefits for patients without compromising patient safety’ (REG3).

#### 3.1.3 Multiple Modalities

Telehealth appointments could be scheduled in advance by optometrists for non‐urgent cases, where the patient returns on a different occasion for the telehealth video consultation with or without additional clinical tests. On‐call telehealth, where optometrists could gain immediate access to ophthalmology, was used for acute cases. The on‐call service was also used opportunistically by visiting optometrists during outreach services in remote locations. This maximised the opportunity of the in‐person interaction and saved the patient, who may have travelled a considerable distance, from having to return for a future scheduled visit.

### 3.2 Outer Setting

#### 3.2.1 Need for Care

The vast distances and limited on‐ground workforce initially drove the use of telehealth. It was described as ‘filling a necessary gap’ (OPTOM2). Insufficient ophthalmology cover to meet needs was exacerbated during COVID‐19 lockdown restrictions and significantly increased the usage and development of this service.

#### 3.2.2 External Stakeholders

Certain barriers to implementation arose relating to the engagement of external stakeholders. Participants explained that due to the federal funding of optometry services, the State government had not generally engaged with optometry. Therefore, few optometrists in Australia practice as part of the state‐funded public hospital health system. Many of the optometrists involved in telehealth were motivated independent practitioners with professional freedom. Some participants reported prior experiences with optometry industry stakeholders with retail‐driven business models which appeared unwilling to incorporate collaborative telehealth into practice.

#### 3.2.3 Funding Model

The LOV funding model for ophthalmology has evolved since its inception in 2011; initially utilising Medicare item numbers, then changing to State government block funding in 2020. This change coincided with statewide telehealth becoming a key component of a regional hub established in the Kimberley region of WA. The public funding for consultants included a roster that shared on‐call telehealth among ophthalmologists to ensure availability and financial sustainability of the service.

For optometry, there was initially no reimbursement, but advocacy resulted in the introduction of Medicare billing codes for telehealth in 2015. Initially, the optometrist was required to be 15 km away from an ophthalmologist to claim the telehealth code, but this requirement was removed during COVID‐19. The reimbursement was tiered for the time spent on the video consultation, though some of the research participants noted this as an inadequate indication of the quality of care. ‘The fact that they have a short and a long [consultation] is indicative of the fact that you can get a shorter version of excellence. And I don't necessarily agree’ (OPTOM2). There were also issues reported with the processing of reimbursements by Medicare, with the system occasionally rejecting the item code, adding another barrier and administrative load for optometrists.

### 3.3 Inner Setting

#### 3.3.1 Equipment

Access to equipment significantly influenced the feasibility and success of telehealth. The ability for optometrists to obtain and transmit test results facilitated the shared decision‐making process undertaken with the ophthalmologist. Examples were anterior photographs, corneal tomography, optical coherence tomography (OCT), widefield retinal imaging and automated perimetry.

#### 3.3.2 Digital Infrastructure

The choice of the video conferencing platform was such that it required no cost investment by practitioners, and was accessible using different digital devices, that is, smartphone, tablet, computer. Participants felt that the interface between optometry and ophthalmology electronic health records was paramount. Shared records allowed practitioners on both sides to view the patient record in real‐time, including patient details, clinical records, booking information and recalls. Participants felt separate systems would also be feasible if the integration of records was possible.

For some participants, image sharing was facilitated by proprietary software that allowed real‐time interaction with scan results by both parties. This was particularly important for equipment such as OCT, where scrolling through multiple retinal scans provided much more information compared to a single cross‐sectional scan. However, due to cost, not all optometrist participants used this, preferring instead to screen‐share or send screenshots through the chat function of the video‐conferencing platform. Internet connectivity was a challenge in some remote regions, though satellite has improved this over time.

#### 3.3.3 Communication and Feedback Loops

Due to the number of people involved in the telehealth model, good communication was required between team members. The ophthalmology telehealth roster helped clinicians understand the appropriate contact person for each day, and group messages helped team members support each other when necessary. Feedback from ophthalmology to optometrists on patient management led to gradual upskilling of optometry and alignment in decision making.

#### 3.3.4 Clinical Setting

Optometrists in different clinical settings had varied challenges. Theoretically, private practice optometrists can charge a private fee outside of the Medicare rebate. However, as telehealth was considered a public ophthalmology service, patients did not expect to pay any out‐of‐pocket fees. For salaried optometrists in a hospital setting, firewalls restricted the choice of software, limiting information and image sharing. In remote outreach settings, internet connectivity issues and limited advanced diagnostic equipment presented challenges.

### 3.4 Individuals

#### 3.4.1 Clinician Capability

The success of this telehealth model depended primarily on the capability of optometrists to perform a thorough eye examination. One optometrist said, ‘there's a lot of trust that the specialist is putting in… they need to feel confident in my assessment…Whatever's been said, you've got to go with [it]’ (OPTOM3). Participants acknowledged there were limitations for non‐eye care practitioners for this type of telehealth model. One participant commented that telehealth ‘can work for GPs…The problem is that if they can't look in the back of the eye, well, then they can't say [if there is] retinal detachment or not’ (OPTOM4).

#### 3.4.2 Ownership and Adaptability

It was important for team members to take ownership and be supportive of telehealth. A registrar commented that at the start of the day, ‘the consultants are very proactive…they'll have a quick flick through [the patient list] and they'll kind of give you suggestions on what they think should be done’ (REG1). Team members were adaptable, ‘we just do whatever's required…there's just no limit to what I'll do to ensure that it works’ (OPTOM2), and supported each other, ‘[if] you're busy or you're with another patient …you can often just message through to that telehealth group chat and say, hey, I'm busy, can someone else pick this up? And someone else will’ (REG1).

#### 3.4.3 Champion

A champion for the service, in this case the lead ophthalmologist, was essential for the success of the model. ‘It's no secret that his passion is ensuring that all of regional WA has access to good tertiary care. And he will be the first one to tell you that optometry is integral in ensuring that this partnership stays alive. And championing optometry from an ophthalmology point of view, I think, ensured that I felt comfortable as a practitioner’ (OPTOM2).

### 3.5 Adoption of Telehealth

#### 3.5.1 Relationship Building

Several members were involved in the coordination and delivery of eye care services in the telehealth model including management, administrative, clinical and ancillary staff. Reciprocal relationships between all members involved in telehealth delivery was key. ‘I feel like we're all in the same team’ (OPTOM1); ‘I don't think the telehealth system would work at all if you didn't have a good working relationship between the ophthalmology service provider and the optometry service provider’ (REG3).

Establishing a relationship between optometry and ophthalmology was important for trust building and communication, critical elements in collaboration. However, participants felt that building relationships took time, and while this facilitated adoption, collaborative models based entirely on relationships are unsustainable when motivated clinicians move on or in the case of a transient workforce.

#### 3.5.2 Optimising Workflow

Practitioners often had a full clinic day, so minimising disruption to workflow was key. Strategies were employed to lower the barrier to entry, such as creating a bespoke booking platform that asked only for key information so as not to be time consuming. Another strategy was using a video‐conferencing platform that has an easy user‐interface. For ophthalmology, the telehealth roster ensured that practitioners were available to provide a timely response to optometry requests for on‐call services.

#### 3.5.3 Role Clarification

Role clarification was important given some overlap in the skills and tasks of the referring practitioner and the ophthalmologist. Optometrists practised to a wide scope within this model, and they reported that being able to do so was fulfilling. However, some optometry participants expressed uncertainty regarding where they fit within the public model, as optometrists were traditionally not involved in public eye care provision in Australia. On the ophthalmology side, a senior ophthalmologist being rostered meant that there was always someone who could be contacted to assume responsibility over the final decision‐making. The responsibility for administrative duties was less clear, with both optometrists and registrars involved in basic triaging and contacting patients.

### 3.6 Future Sustainability

#### 3.6.1 Patient Expectations

It was important to set patient expectations in line with the model to ensure they understood the multiple decision makers involved in the process. Participants described the patient experience as being extremely positive regarding clinical outcomes and communication; however, it should be noted that patients were not directly interviewed in this study.

#### 3.6.2 Staff Availability

Having sufficient staff available to triage referrals and schedule patients and optometrists and ophthalmology service providers willing to collaborate for telehealth was important for sustainability.

#### 3.6.3 Cultural Safety

There is a high proportion of Aboriginal and Torres Strait Islander people who live in rural and remote areas. Participants felt cultural safety had a profound impact on the trust developed between communities and providers, on attendance rate and utilisation of services, and subsequently on patient health outcomes. A key advantage of telehealth was allowing patients to remain close to home to access care on Country. The local optometrists, who are often well‐known within the community, provide a stable point of care for patients. ‘Often [when] Aboriginal people don't understand something they will say “yes” because they don't want to be rude… whereas having the optometrist there… [they] know how to make it more culturally appropriate for them to understand’ (NON‐C1).

### 3.7 Intersection of Sustainability and Scalability

#### 3.7.1 Stakeholder Buy‐In

Continued buy‐in from already involved practitioners was required for the model to be sustainable. For scalability, the relative advantage of telehealth needs to be showcased to obtain buy‐in from practitioners who could use the service, as well as government and funders who make decisions that ultimately impact how feasible service expansion will be. Participants felt that broader awareness of the relative advantage of telehealth will be necessary for increased uptake.

#### 3.7.2 Economic Considerations

Continued support for resource allocation and renumeration was identified as a key driver for the sustainability of the service. Participants felt the service needed to be sustainable given the significant benefits from a government and societal standpoint. ‘If you look at it from an economic perspective, it just makes sense for the government to remunerate it because one [patient] transfer costs upwards of $1000 plus’ (REG3). To achieve this, ophthalmology requires ongoing funding and support for sufficient workforce levels. Optometrists perceived current Medicare reimbursement amounts to be lower than sustainable, leading to a large difference between telehealth and that of usual private practice. One optometrist explained, ‘I think if we run the numbers over the last five years…as a comparative analysis against if I had dispensed glasses at the rate at which we dispense…I think that you would see that we probably lose money, but I think that in general we have a better quality of practice life and we're far better plugged into the community’ (OPTOM2). Although some participants were willing to overlook the financial drawbacks of telehealth due to its other benefits, the long‐term sustainability and potential expansion of this model remain uncertain.

#### 3.7.3 Telehealth‐Suitable Conditions

Although there was no consensus on a definitive list of conditions that are ‘suitable’ for telehealth, participants recognised that conditions such as cataract and pterygium could be safely and successfully waitlisted for surgery, given that the optometrist performs a thorough examination to identify risk factors for complicated surgery. Chronic conditions identified as suitable for telehealth included diabetic retinopathy, age‐related macular degeneration, and glaucoma. Cases with high quality images that could be transmitted were also useful. Participants agreed that face‐to‐face care, rather than telehealth, was most beneficial for undifferentiated cases where the examining optometrist was unsure of the diagnosis.

#### 3.7.4 Artificial Intelligence

Participants described multiple future perceived roles of artificial intelligence (AI) in telehealth including triage and appointment management, scribing for clinical documentation, and diagnostic support. Participants felt that the deep‐learning capabilities of AI might enhance the quality of triaging over time. The potential to use AI‐scribing to summarise any technical jargon spoken between providers into a patient report was also discussed.

#### 3.7.5 Asynchronous Telehealth

Participants identified the potential for asynchronous telehealth to improve the efficiency of the current model. The patient would not need to be physically present, and clinical findings can be reviewed at a convenient time, which may yield time efficiencies for both practitioners and patients. Examples provided of suitable chronic conditions included glaucoma and diabetic retinopathy. Similar models could also empower non‐eye care trained professionals such as general practitioners to utilise modern cameras and AI tools to screen for systemic diseases, such as renal and cardiovascular disease. Engaging in opportunities for telehealth with different professions can help to achieve universal eye care coverage.

#### 3.7.6 Public Health Infrastructure

The integration of telehealth into public health infrastructure was a recognised opportunity to scale this programme. A well‐resourced tertiary institute could aid long‐term sustainability due to a larger workforce. One challenge would be the use of technological platforms in state‐based health systems, which participants felt could be restrictive.

### 3.8 Scalability of the Model

#### 3.8.1 Regulation

As with other digital health initiatives, it is essential to ensure the security of health data collection and to ensure all platforms are in line with privacy laws and policies. Participants noted that any use of AI will need to meet regulatory guidelines at the time of implementation.

#### 3.8.2 Telehealth Guidelines

Participants recognised that for broader scalability of the model, there would likely be a need for standardisation of telehealth through recommendations or guidelines. The development of any guidelines would require flexibility for different geographic, cultural and health service contexts. For example, due to the different surgical facilities in each state, having an interstate optometrist booking surgery through a Western Australian telehealth service was unlikely to be feasible. Participants felt that useful guidelines should include types of conditions that are appropriate for telehealth, investigations that are necessary for certain conditions, and ethical, privacy and regulatory considerations. However, participants noted that restrictive guidelines can potentially lead to lower practitioner uptake of the service or have the potential to make processes inefficient.

#### 3.8.3 Training

The genuine collaboration between optometry and ophthalmology and upskilling of clinicians were strengths of this model. In‐person interactions when ophthalmologists visited local clinics allowed opportunities for training and gradual trust‐building to occur. Optometrists reported that engaging with public ophthalmology services required a shift in mindset from primary care to secondary care. It was suggested that any scaling of this model could be accompanied by training to educate optometrists about secondary and tertiary eye care decision‐making and surgical considerations.

#### 3.8.4 Incentive

Participants felt that incentives are required to increase uptake of telehealth services. Optometrists involved in this model perceived there to be almost no current financial incentives to conduct telehealth. ‘The [optometry] clinic runs at a loss to provide a public service… it would be a far more viable thing to do if Medicare remunerated disease management properly’ (OPTOM4). Rather than a one‐off incentive, participants suggested ongoing incentives for sustained implementation. For example, if a telehealth Medicare code is billed every quarter, then a financial incentive could be provided that could contribute towards digital infrastructure for telehealth. Similarly, an incentive would be required to motivate ophthalmologists to collaborate. Other than financial, education and training incentives could also motivate practitioners to participate in telehealth.

#### 3.8.5 Metropolitan Areas

Although LOV telehealth has primarily serviced rural areas, participants expressed that the model could be applied in any context where the system is unable to keep up with the demand for care, including metropolitan and inner‐regional areas. One such opportunity was triaging suitable referrals into satellite optometry clinics outside of the hospital, which could provide more space for complex conditions within the public hospital system. Expedited surgical bookings could also benefit metropolitan and regional areas. Participants proposed that data‐informed strategies could be used to trial expedited care in areas with population‐based need. Data on population size, age, disease status, existing access to care and socioeconomic advantage can reveal areas where telehealth would be of most benefit.

## 4 Discussion

The interviews explored in‐depth the contextual factors that have led to sustained implementation of this interprofessional collaborative telehealth model for eye care. The study highlights the potential for optometry to reduce public ophthalmology burden and demonstrates how existing healthcare resources can be more effectively utilised through collaboration to achieve better outcomes. Evaluating initiatives supports the development of evidence‐driven models and allows for their systematic integration into the broader health system [30]. The main findings that highlighted why the LOV telehealth programme has been successful to date were the existing need for care, relative advantage of the model, choice of digital infrastructure, relationships, consideration for workflow optimisation, team member adaptability, and a champion. This study found several unique barriers and enablers specific to collaborative eye care, such as the importance of workflow optimisation for clinicians on both sides, the importance of triage and inadequate bulk‐billing rates and incentives to promote uptake of telehealth. The results indicated a clear need for a systems approach and data‐informed strategies for sustainability and scalability of the model.

Our study findings suggest that new guidelines to support collaborative telehealth practices in Australia would be beneficial. The Optometry Australia Telehealth Clinical Practice Guide [31] was published in 2021 and primarily focuses on teleoptometry. The guideline does not include coordinated telehealth practices with ophthalmology nor insight into telehealth‐suitable conditions. As indicated by the participants in the study, chronic disease management is well‐suited for telehealth, as is expediting surgery through direct bookings and management of acute cases where the diagnosis is certain. The lessons learned from this model could form the foundation for a set of guidelines that could be adapted for broader use.

A challenge to collaborative telehealth is the logistical and financial burden associated with coordinating real‐time consultations between two busy clinicians. Asynchronous telehealth for stable conditions presents a promising solution, particularly when supported by appropriate and scalable funding structures. Indeed, much of the existing literature on telehealth in eye care has focused on asynchronous diabetic retinopathy, glaucoma and retinopathy of prematurity screening [32]. Australian models like the C‐EYE‐C for glaucoma [33] and diabetes [34] have also demonstrated reduced wait times and cost savings of between 22% and 43% for the health system through task shifting. The combination of asynchronous, synchronous and in‐person assessments could potentially streamline the process and improve the overall efficiency of the healthcare system as in other professions such as psychiatry [35] and dermatology [36].

For funders, there may be concern that investment in telehealth may not present as a short‐term cost‐saving for the healthcare system, despite the longer term benefits of improved health outcomes [37]. However, previous modelling has demonstrated that investment in telehealth can be offset by significant savings from avoiding patient transfers [38]. Moreover, optometrists already have suitable equipment for ocular imaging established, compared with other potential teleophthalmology collaborators such as general practitioners [38]. Additionally, for conditions like cataract where long wait times can result in increased risks from falls or decreased independence due to driving cessation [39], timely intervention provided by telehealth could have significant flow‐on social and economic benefits. Given existing financial constraints in healthcare systems worldwide, adopting a long‐term perspective is essential. Further research is required to evaluate the economic impact of this model compared to traditional face‐to‐face care models and assess the necessary reimbursement levels for sustainability.

A limitation of this study is that only the perspectives of healthcare staff were explored, so any patient perspectives were second‐hand. However, patient perspectives of this particular service have been previously explored, where patients also reported satisfaction with the familiarity of a local optometrist who could assist with the technological aspects of telehealth [9]. Furthermore, as the telehealth model explored in this study is specific to Western Australia, findings may not be generalisable to other regions or healthcare systems. However, detailed descriptions of the setting and participants were provided for context, and the findings provide a strong basis for further exploration in different geographic and healthcare settings. Future research could explore the attitudes and perceptions of practitioners not currently involved in telehealth, to understand barriers to adoption and identify strategies to facilitate broader integration of telehealth into practice. Improved interoperability of systems and AI will further facilitate the adoption of innovative modes of digital health; however, economic considerations and regulation appear necessary [40].

## 5 Conclusions

The results of this study highlight the impact that workforce utilisation, collaboration and harnessing digital health can have on access to eye care. Technological infrastructure and systems interface are vital for collaborative telehealth. Champions, flexibility and adaptability of team members were crucial to the success of this model. The development of guidelines that can be adapted to suit local context and population needs may further enhance the scalability of this model. As digital health innovations evolve, it will be important that education, training, legislation and appropriate remuneration evolve concurrently to reflect modern healthcare provision.

## Author Contributions

**Sharon A. Bentley:** writing – review and editing, conceptualization. **Stephen E. Bartnik:** writing – review and editing. **Jingyi Chen:** conceptualization, investigation, writing – original draft, formal analysis, writing – review and editing. **Allison M. McKendrick:** conceptualization, writing – review and editing. **Khyber Alam:** conceptualization, formal analysis, writing – review and editing. **Angus W. Turner:** conceptualization, writing – review and editing, formal analysis. **Sandra C. Thompson:** conceptualization, writing – review and editing.

## Funding

The authors have nothing to report.

## Ethics Statement

The study was performed in accordance with the Helsinki declaration. Ethical approval was granted by the University of Western Australia Human Ethics Committee (2024/ET000213).

## Consent

All participants provided informed consent before participating in the study.

## Conflicts of Interest

The authors declare no conflicts of interest.

## Supporting information


**File S1:** Interview guide for the evaluation of a collaborative telehealth model for eye care in Western Australia.

## Data Availability

The datasets used and analysed during this study are not shared publicly for privacy and ethical reasons. They may be available from the corresponding author on reasonable request.
