# Supplementary material for: Evaluation of a Collaborative Telehealth Model for Eye Care Between Ophthalmology and Optometry in Western Australia
Source: Aust J Rural Health. 2026 May 7;34:e70203. doi: 10.1111/ajr.70203 (PMC13150471; doi:10.1111/ajr.70203)
Supplement: Supplementary file 1 — File S1: Interview guide for the evaluation of a collaborative telehealth model for eye care in Western Australia. [file AJR-34-0-s001.docx]

**Supplementary File: Interview guide for the evaluation of a collaborative telehealth model for eye care in Western Australia**

Thank you for agreeing to participate in our study. We really appreciate your time.

Before we begin, I would like to check that you are still happy to take part in the study. I would like to confirm that we have received the signed consent form and would also like to confirm you’re happy for this session to be recorded.

*Start recording*

The aim of this study is to understand the attitudes, perspectives, and experiences of the rural Western Australia eye care workforce. We have asked you to participate in this interview as you have experience in telehealth for eye care.

There are no right or wrong answers in the interview, and you do not have to answer certain questions if you would prefer not to. However, I am interested in hearing about your experiences so please feel free to go into as much depth as you’d like. This interview is confidential. We may use quotations from the transcript, but this will be de-identified so your name will not appear anywhere.

If at any point during the interview you would like to take a break or stop, please let me know.

Do you have any questions before we begin?

Thank you. We will now move on to the interview.

| **General**  First, I would like to ask you about your involvement and experience with telehealth for eye care. |
| --- |
| - Tell me about the telehealth model you are involved in, how does it work? - Tell me about your role in the telehealth model. - When and how did you first get involved in this telehealth model? - (For clinicians) How many years of clinical experience do you have? - In your opinion, how do you think this telehealth model compares to a traditional face-to-face model for ophthalmology services? (Prompts: Patient and clinician perspective) - What works well and what doesn’t? What could be improved? |
| **Individuals’ domain**  Next, I would like to ask you some questions about the motivation and the people involved in the telehealth model. |
| - What are the factors that have motivated you to continue using telehealth? - What influence do the relationships between team members have on the success of telehealth? |
| **Inner setting domain**  Now I would like to ask you some more questions about the telehealth model. |
| - What processes or systems are in place that help with successful communication between team members? - How well does this telehealth model fit in with your usual day to day practice? (Prompts: e.g. running telehealth within a private optometry practice or in a hospital, or as part of a standard day of ophthalmology appointments) - How much of a priority is placed on telehealth within the day? - Can you tell me about the technology and IT infrastructure that is required for telehealth? (Prompts: connectivity, internet, videoconferencing software, electronic documentation, data storage, security, privacy, upkeep, support) Does this differ depending on the location? Have you faced any technological challenges in telehealth? - How important is advanced diagnostic equipment for the delivery of telehealth? - How well-defined are the responsibilities amongst the team? How important is defining responsibilities? (Prompts: i.e. optometry and ophthalmology and admin) - Have there been any incentives involved in the program, financial or otherwise? - Has there been any formal or informal training of team members for telehealth? - If yes: What training has been done to ensure the team was able to implement this model effectively? - If no: What training would be helpful? |
| **Implementation process domain**  **Thinking back to when you first started using telehealth for eye care,** |
| - What barriers did you experience? - What strategies were employed to overcome the barriers that you have just mentioned? - How successful were they? |
| **Innovation domain** |
| - Do you think the telehealth model could be applied elsewhere? Why or why not? (e.g. in a metropolitan area) (Prompts: discuss levels of collaboration in metro areas) - Are you aware of any guidelines for this type of telehealth? - How important is it that guidelines or a framework are established and followed in collaborative telehealth? What sort of guidelines (or framework) would be useful? (Prompts: types of conditions that can be managed using telehealth, at which point is F2F care required) - How adaptable would these guidelines (or this framework) be if this model was replicated in a different setting? (Prompt: type of practice, different location, level of comfort of practitioners) Are there components that should not be altered? |
| **Outer setting domain** |
| - Has there been any external pressure to implement telehealth? (Prompts: other types of external pressure include societal pressure, market pressure, performance-measurement pressure, corporate pressure) - Have there been any policies or regulations that have enabled or limited aspects of this telehealth model? (Prompts: workplace, professional regulation) - What funding has this service received? (Prompts: Clinical and administrative) - How important is funding for the long-term sustainability of this model? |
| **Future and sustainability**  I’d like to explore what the future potentially looks like for telehealth. |
| - If everything was to continue as it is, how sustainable would this model be in the future? - What factors would be important to help support the sustainability of this model? - Are there any factors that would risk or negatively impact the sustainability of this model of telehealth? - Can you see the role of artificial intelligence in telehealth? |
| **Asynchronous telehealth**  Asynchronous telehealth is the process whereby communication between practitioners is not live, and clinical records can be forwarded and reviewed at a later time. |
| - Have you used asynchronous telehealth before? Tell me about your experience with it. - If no: Is asynchronous telehealth a model that you would consider using?   What are the perceived benefits or challenges to asynchronous telehealth? |

Is there anything that you would like to add, that we haven’t covered?

Thank you for contributing to this important research – your contribution is very much appreciated.
